# Crystal Structures of Archaemetzincin Reveal a Moldable Substrate-Binding Site

**DOI:** 10.1371/journal.pone.0043863

**Published:** 2012-08-24

**Authors:** Christine Graef, Magdalena Schacherl, Sandro Waltersperger, Ulrich Baumann

**Affiliations:** Department of Chemistry and Biochemistry, University of Bern, Bern, Switzerland; University of Oulu, Finland

## Abstract

**Background:**

Archaemetzincins are metalloproteases occurring in archaea and some mammalia. They are distinct from all the other metzincins by their extended active site consensus sequence HEXXHXXGXXHCX_4_CXMX_17_CXXC featuring four conserved cysteine residues. Very little is known about their biological importance and structure-function relationships.

**Principal Findings:**

Here we present three crystal structures of the archaemetzincin *Af*AmzA (Uniprot O29917) from *Archaeoglobus fulgidus*, revealing a metzincin architecture featuring a zinc finger-like structural element involving the conserved cysteines of the consensus motif. The active sites in all three structures are occluded to different extents rendering the enzymes proteolytically inactive against a large variety of tested substrates. Owing to the different ligand binding there are significant differences in active site architecture, revealing a large flexibility of the loops covering the active site cleft.

**Conclusions:**

The crystal structures of *Af*AmzA provide the structural basis for the lack of activity in standard proteolytic assays and imply a triggered activity onset upon opening of the active site cleft.

## Introduction

Zinc-dependent endoproteases are involved in many essential biological processes like protein degradation and thus regulation of the metabolism [1]. Many of these enzymes belong to the MEROPS clan MA [2] and are characterized by a conserved consensus sequence, HEXXH, where the two histidines serve as ligands for the metal ion and the glutamic acid acts as catalytic base polarizing a zinc-bound water molecule for nucleophilic attack on the peptide bond of the substrate [3], [4].

The metzincins constitute subclan MA(M) [2] of these zinc-dependent proteases and comprise, besides others, the families of the astacins, ADAMs/adamalysins, serralysins, matrix metalloproteinases, leishmanolysins, snapalysins, pappalysins and the archaemetzincins [5]. All the metzincins share a common catalytic domain architecture of about 130 to 260 residues consisting of an N-terminal and C-terminal subdomain divided by the active-site cleft [6]. The N-terminal domain exhibits a twisted, mainly parallel β-sheet and two helices, the backing helix and the active site helix. In contrast to other metalloproteases, the active site is characterized by an extended consensus sequence HEXXHXXGXX(H/D) [5]–[8]. The third zinc-ligand is the side chain of a histidine or aspartate moiety three residues downstream of a strictly conserved glycine. The name of this family is derived from a structurally and spatially conserved 1,4-β-turn found directly below the zinc binding site in the C-terminal domain comprising a methionine at position three in the ß-turn in all identified metzincins. Methionine-replacement studies of protease C (PrtC) from *Erwinia chrysanthemi* [9], [10] and ulilysin from *Methanosarcina acetivorans* [11] emphasized the importance of this residue for the structural and functional integrity of the active site.

Archaemetzincins (MEROPS family M54.001) are a hitherto only scantily characterized protease family occurring mainly in archaea but also in higher mammals and very few eubacteria. Structural and functional information is sparse, mainly deriving from a member from *Methanopyrus kandleri* [12] and an unpublished crystal structure of a hypothetical protein from *Methanocorpusculum labreanum* [13].

In addition to the structural features characteristic for all metzincins, the archaemetzincins display four conserved cysteine residues downstream of the active site consensus sequence which were found to bind zinc [12] or allegedly iron ions [13]. Despite the presence of all known elements necessary for an active proteolytic enzyme, no activity against typical *in vitro* exo- and endopeptidase substrates has been detected so far for the *M. kandleri* enzyme [12]. This could imply a very stringent substrate specificity of the archaemetzincins or the need for additional activating factors.

Vertebrates possess homologs with a similar core domain sharing some 25% sequence identity, e.g. in the human archaemetzincin-1 and -2 (AMZ1 and AMZ2) [14]. These enzymes are found in various fetal and adult tissues and have been described as aminopeptidases with high specificity for alanine (AMZ1) and arginine (AMZ2). However, AMZ1 has the third histidine of the metzincins' consensus sequence replaced by asparagine, serine or threonine, depending on the organism. These amino acids are not known to function as zinc ligands, thus raising some concern on the proteolytic activity of AMZ1. Similarly, in CarG, a bacterial homolog from *Myxococcus xanthus* the catalytically essential glutamic acid of the HEXXH motif is replaced by a glutamine residue [15]. Here however, this archaemetzincin has a well-established function as a subdomain of a transcriptional regulator and is proteolytically inactive in accord with that mutation.

In order to expand our knowledge on the structure-function relationships of the archaemetzincins we have determined the crystal structure of native and tagged archaemetzincin (UniProt entry O29917) from *Archaeoglobus fulgidus* (*Af*AmzA) in three different crystal forms to 1.40 Å and 2.16 Å resolution, respectively. Like AmzA from *Methanopyrus kandleri*, *Af*AmzA possesses all features of proteolytcially active metzincin proteases. However, the protein from *M. kandleri* did not exhibit any detectable proteolytic activity in our assays and displayed a closed active site cleft [12].

## Results and Discussion

Recombinant *Af*AmzA was produced in *E. coli* as a native, untagged (referred to as nat-*Af*AmzA) and as an N-terminally 6xHis-tagged version, respectively. The 6xHis-tag was removed by thrombin yielding the full-length protein with three additional residues preceding the start methionine (referred to as NHis-*Af*AmzA.).

### Overall structure

**Figure 1:**
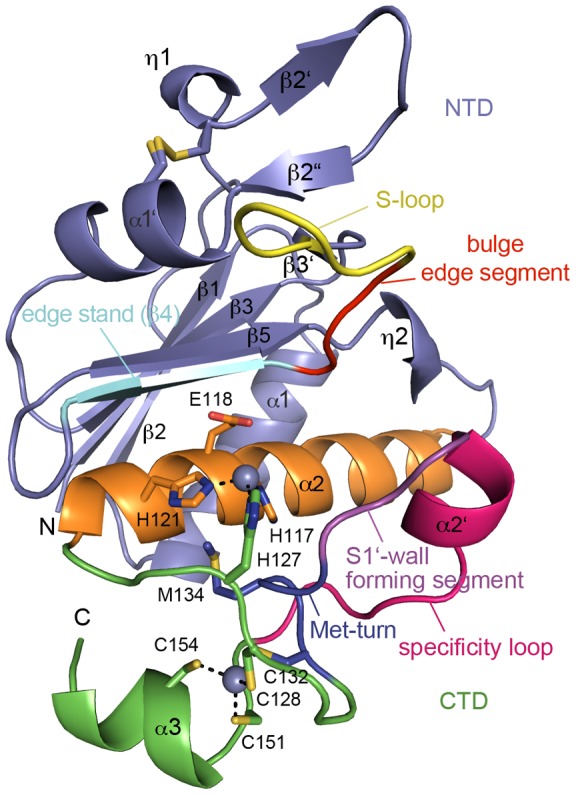
Structure of *Af*AmzA. Overall structure of *Af*AmzA in cartoon representation. The N-terminal domain (NTD) is colored in slate, the active site helix α2 in orange and the C-terminal domain (CTD) in green. The N- and C-termini, the edge strand β4 (cyan), the backing helix α1, the S-loop (yellow), the bulge edge segment (red), the S1′-wall forming segment (magenta) and the specificity loop (purple) are labeled. The residues involved in zinc ion binding, the catalytic base and the structurally important methionine are shown as sticks and the zinc ions as spheres.

**Figure 2:**
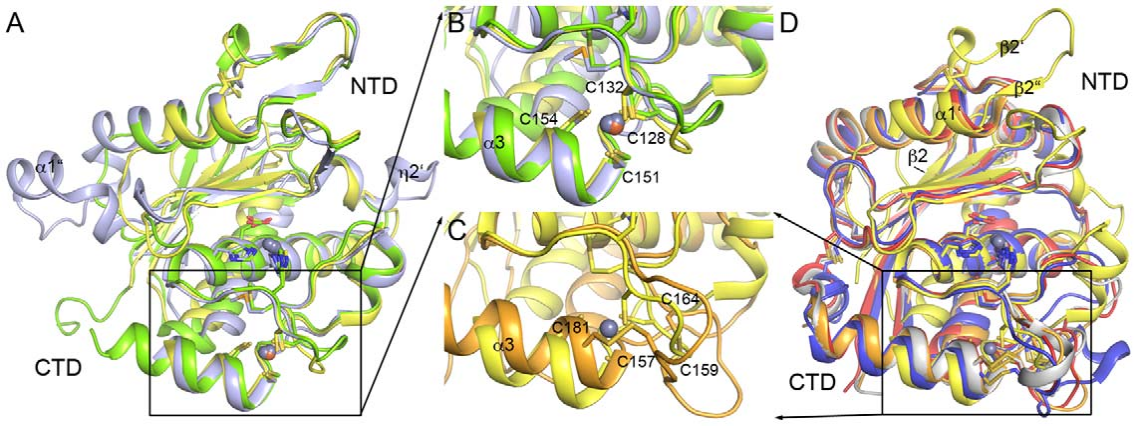
Structural alignment of archaemetzincins and metzincins. (A) Superposition of the three known AmzA structures *Af*AmzA (yellow), *Mk*AmzA (green) and *Ml*AmzA (slate). (B) Close-up view of the Cys_4_ zinc finger domains of all three AmzA structures from (A). (D) Superposition of *Af*AmzA (yellow) with selected metzincins Bap1 (orange), H2-proteinase (grey), acutolysin A (red) and ADAM33 (blue). In place of a Cys_4_ zinc finger found in *Af*AmzA (yellow), the superposed metzincins exhibit two disulfide bonds as shown in (C) using the example of Bap1 (orange). Individual cysteine residues are labeled in accordance with the amino acid sequence of *Af*AmzA (yellow) in (B) and Bap1 (orange) in (C), respectively. Important secondary structure elements are labeled.

**Figure 3:**
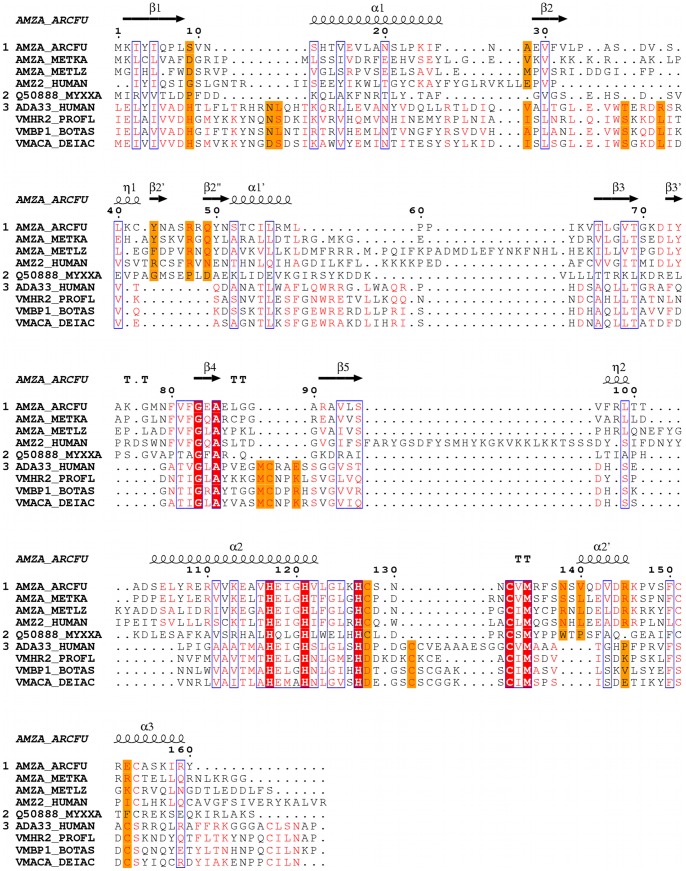
Multiple sequence alignment of the amino acid sequences of *Af*AmzA with other archaemetzincins and metzincins. Archaemetzincins from *M. kandleri* (AMZA_METKA), *M. labreanum* (AMZA_METLZ), *H. sapiens* (AMZ2_HUMAN), non-catalytic archaemetzincin from *M. xanthus* (Q50888_MYXXA) and metzincins *H. sapiens* ADAM33 (ADA33_HUMAN), *P. flavoviridis* H2-proteinase (VMHR2_PROFL), *B. asper* Bap1 (VMBP1_BOTAS) and *A. acutus* acutolysin A (VMACA_DEIAC). Sequences were aligned using Chimera [38], [39] and visualized with ESPript [40]. 3_10_-Helices are indicated by η, β-turns by TT. Conserved residues in all sequences are highlighted in red. Similar sequences are in red letters, orange color indicates residues similar in each group (1, 2 or 3) but significantly different from the other groups.

The overall structure of *Af*AmzA (Fig. 1) resembles those of other metzincins [6], consisting of an upper N-terminal domain (NTD) and a lower helical C-terminal domain (CTD) with respect to the active site helix α2, which harbors two of the three zinc-binding histidines (H^117^EIGH^121^) and the catalytic base Glu118. The NTD is composed of a twisted five-stranded β-sheet, the backing helix α1 and a few additional elements, like two short 3_10_-helices η1 and η2 as well as a second small β -sheet (strands β2′-2′′-3′) connecting the main secondary structure elements. Furthermore it accommodates the edge stand β4 and the bulge edge segment involved in substrate recognition and binding. The S-loop, which is engaged in calcium- and zinc-ion binding in some other metzincins [16], remains uncomplexed in *Af*AmzA. On the other hand, as the homologous archaemetzincins from *Methanopyrus kandleri* (*Mk*AmzA) [12] and Methanocorpusculum labreanum (MlAmzA) [13], *Af*AmzA exhibits a second zinc-binding site located in the CTD immediately below the catalytic zinc binding site and close to the eponymous, structurally important Met-turn [10]. This element, also named Cys_4_ zinc finger (Cys_4_-Zn), is composed of the four conserved cysteine residues included in the archaemetzincin fingerprint sequence HEXXHXXGXXHC^128^X_4_C^132^XMX_17_C^151^XXC^154^ (Fig. 1, 2*B*, 3).

Opposite of the bulge edge segment the CTD harbors further determinants of substrate specificity, namely the S1′-wall forming segment and the specificity loop [17]. Structural superimposition of all three known archaemetzincin structures (Fig. 2*A*) depicts a very high overall structural similarity within the members of the archaemetzincin protease family (*Af*AmzA rmsd to *Mk*AmzA is 1.5 Å and to *Ml*AmzA is 1.7 Å for 155 Cα atoms).

In order to compare the archaemetzinincs and especially *Af*AmzA to other metzincin families, the structure was overlayed on Bap1 (PDB code 2W14, rmsd 2.6 Å), H2-proteinase (1WNI, rmsd 2.4 Å), acutolysin A (1BSW, rmsd 2.2 Å) and ADAM33 (1R54, rmsd 2.4 Å) (Fig. 2*D*). Except for the short insertion (η1-β2′-β2′′) between strand β2 and helix α1′ present in *Af*AmzA, the NTDs of all five structures superpose well. Structural similarity is also found within the catalytic zinc-binding site and the Met-turn, while significant differences are observed within the cysteine-rich CTD. As described above, archaemetzincins contain a Cys_4_ zinc finger (Fig. 2*A, B*) formerly believed to be involved in disulfide bond formation [18], whereas in the other metzincins mentioned above, four cysteines located at similar positions form two disulfide bridges instead (Fig. 2*C, D*, *3*).

### The active sites of NHis-A*f*AmzA and nat-A*f*AmzA

**Figure 4:**
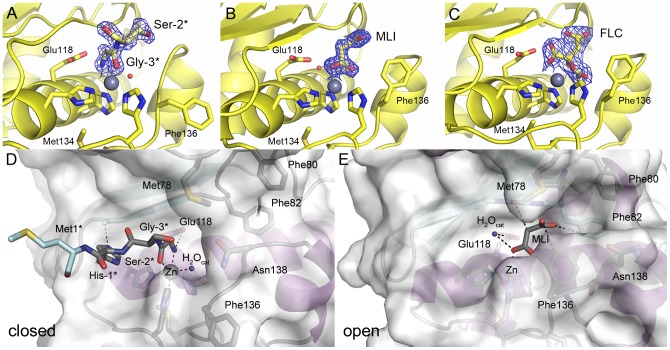
Structural differences between NHis-*Af*AmzA and nat-*Af*AmzA. Simulated annealing F_o_–F_c_ omit map of (A) the N-terminus of a symmetry-related molecule (Gly-3*-Ser-2*) in NHis-*Af*AmzA, (B) a malonate molecule (MLI) in nat-*Af*AmzA::malonate and (C) a citrate molecule (FLC) in nat-*Af*AmzA::citrate, contoured at 2σ level. The maps were generated using phenix.omit_map [37] and converted to the ccp4 format with FFT (V6.1) [32]. Important residues are shown as sticks. The zinc ions and water molecules are shown as grey and red spheres, respectively. (D) Surface representation of NHis-*Af*AmzA active site cleft with the N-terminus of a symmetry related molecule bound to the catalytic zinc ion (primed site in closed conformation). (E) Surface representation of nat-*Af*AmzA active site cleft with the zinc-bound malonate molecule (MLI; primed site in open conformation). Important residues and the catalytic water molecule (H_2_O_cat_) are labeled. The surface is transparent to allow a view on the residues involved in zinc ion and ligand binding.

The crystal structures of NHis-*Af*AmzA and nat-*Af*AmzA show rather large differences in the substrate-binding site. The first three amino acids of the NHis-*Af*AmzA construct employed in this study are the remainder of the N-terminal 6xHis-tag after thrombin cleavage and will be denoted by negative residue numbers, thus as Gly-3, Ser-2 and His-1. In the NHis-*Af*AmzA crystal structure, the catalytic zinc ion is coordinated by the very N-terminal glycine residue Gly-3* of a crystallographic symmetry-related molecule (Fig. 4*D*) leading to an octahedral coordination geometry with His117, His127, Gly-3*(O) and Gly-3*(N) in one plane and a water molecule and His121 in the other plane. By the bidentate coordination of the (deprotonated) amino terminus and the carbonyl oxygen of Gly-3* the zinc-bound water is displaced from its spatial position between the zinc ion and the catalytic base. Such crystal contacts have also been found in inhibitor-free ADAM33 [19]. Furthermore, the metal-ligand interactions are similar to those observed in crystal structures of metzincins complexed with cognate proteinaceous inhibitors such as serralysins [20], [21] or MMPs [22]. Contrary to substrates, the artificial N-terminal overhang is positioned in a parallel manner to the edge strand ß4 and does not form backbone-backbone interactions with this substrate-fixing element. This is not necessarily a consequence of the parallel orientation as has been demonstrated for the pro-peptide of astacin, which is positioned in a parallel manner as well and where two backbone hydrogen bonds between the pro-peptide and the edge-strand are formed [23]. Instead, in the crystal lattice of NHis-*Af*AmzA side-chain hydrogen bonds are found between His-1*(Hε2) and Ala85(O) of the edge-strand β4 as well as Gly-3*(H1) and Glu118(Oε2) (Fig. 4*A, D*).

Obviously, the natural polypeptide chain would start earliest at Met1, which does not contact a symmetry equivalent molecule and therefore this interaction is a crystal artifact. However, this contact has effects on the unprimed and primed substrate binding sites as inferred from comparison with the other crystal forms of *Af*AmzA reported here.

In the untagged nat-*Af*AmzA the artificial three amino acids at the very N-terminus of NHis-*Af*AmzA are missing and that particular crystal contact described above cannot be formed. Consequently, two different crystal forms are observed, one triclinic and one hexagonal. Despite different space groups and solvent contents (Table 1), the conformations of the molecules in the new crystal forms are very similar to each other but at the same time they are substantially different from the original tetragonal form (NHis-AfAmzA) with respect to their active-site binding clefts. The pairwise mean RMS difference between the three crystal forms is about 0.4 Å for 158 Cα atoms as calculated by RAPIDO [24] (Fig. S1).

**Table 1:** Data collection and refinement statistics.

	NHis-*Af*AmzA	nat-*Af*AmzA::malonate	nat-*Af*AmzA::citrate
*Data collection*			
Space Group	I4	P1	P6_3_22
Cell dimensions			
a, b, c, [Å]	88.34, 88.34, 50.28	35.30, 63.49, 64.53	111.93, 111.93, 102.09
α, β, γ [deg]	90, 90, 90	71.39, 86.68, 82.67	90, 90, 120
Protomers per a.s.u.	1	3	1
Solvent Content [%]	54	51	76
Resolution [Å] (highest resolution bin)	25.40–1.40 (1.48–1.40)	31.54–1.40 (1.48–1.40)	49.07–2.16 (2.29–2.16)
No. measurements	155576 (24079)	211457 (31473)	268780 (42298)
Unique reflections	38203 (6043)	96247 (14557)	20756 (3233)
Completeness [%]	99.5 (98.6)	91.8 (85.8)	99.8 (98.7)
Rsym‡ [%]	4.9 (68.8)	3.4 (24.2)	9.6 (88.5)
<I/σ(I)>	15.77 (1.99)	13.6 (3.08)	17.2 (2.72)
*Refinement Statistics*			
Resolution [Å] (highest resolution bin)	25.40–1.40 (1.45–1.40)	31.54–1.40 (1.45–1.40)	48.47–2.16 (2.27–2.16)
Rwork/Rfree§ [%]	16.4/18.6	14.4/16.4	19.4/22.0
Reflections	38202	96244	20753
*RMS deviations*			
Bond lengths [Å]	0.008	0.012	0.008
Bond angles [deg]	1.230	1.434	1.067
*Average B factor [Å^2^]*			
All protein atoms	16.50	15.6	43.4
Waters	32.43	28.0	45.6
Metal ions	13.53	11.4	34.1
Ligands	-	14.7	34.6
*Ramachandran plot % * [*41*]			
Most favored	100.0	100.0	100.0
Allowed	0.0	0.0	0.0
PDB entry	4AXQ	3ZVS	4A3W

‡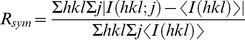 with I(hkl; j) is the jth measurement of the intensity of the unique reflection (hkl) and 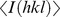 is the mean over all symmetry-related measurements.

§ Random 5% of working set of reflections [42] with 
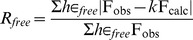
 and 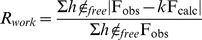.

In contrast to the substrate-binding groove of tetragonal NHis-*Af*AmzA (Fig. 4*D*) which is occluded by the side chains of Met78 and Asn138, the primed side of nat-*Af*AmzA in triclinic and hexagonal crystals provides enough space to accommodate a malonate (Fig. 4*B, E*) or citrate molecule (Fig. 4*C*), respectively. These carboxylic acids originate from the mother liquor of the crystals and coordinate the catalytic zinc ion. In the nat-*Af*AmzA::malonate complex the catalytic zinc ion is coordinated in a tetrahedral geometry by the three histidine side-chains as well as by one carboxylate oxygen atom (O7) of malonate, with ligand-metal distances of 2.0–2.1 Å. The catalytic water molecule is displaced by the malonate O7 atom and is moved towards the catalytic base Glu118. In the nat-*Af*AmzA::citrate complex the catalytic zinc ion is penta-coordinated in a either slightly distorted square pyramidal or a bidentate tetrahedral geometry [25] by three histidine imidazoles as well as by the carbonyl oxygen (Oβ1) and hydroxyl oxygen (OHβ) of citrate, with ligand-metal distances of 2.0–2.2 Å. The catalytic water molecule is completely excluded from the zinc environment. In the case of citrate as zinc ligand, the S1′-wall forming segment has to move to avoid steric clashes with the *pro-S* -CH_2_-COO- branch of the ligand. This has also been observed in a citrate-bound crystal form of the related *M. kandleri* archaemetzincin (Fig. S2).

**Figure 5:**
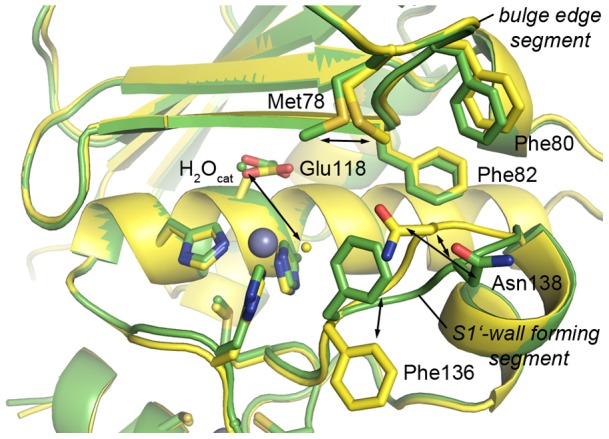
The two conformations of *Af*AmzAs substrate-binding site. Superposition of NHis-*Af*AmzA (yellow) and nat-*Af*AmzA (green) substrate binding site. Changes in the position of side chains in the bulge edge segment (Met78, Phe80,82), side chains and the main chain of the S1′-wall forming segment (Phe136, Asn138) and in the position of the catalytic water molecule (H_2_O_cat)_ are indicated by the arrows.

Besides acting as zinc ligands, these carboxylic acids also mediate a new crystal contact consisting of a salt bridge between one of their carboxylate groups and Arg152 of a symmetry-related molecule. As a consequence, significant differences are observed between NHis-*Af*AmzA and the two carboxylate-coordinated nat-*Af*AmzA crystal forms. While both nat-*Af*AmzA structures with carboxylic acids bound in the active-site cleft superpose quite well (Fig. S1) despite different crystal lattices, the structural superimposition of nat-*Af*AmzA::malonate with NHis-*Af*AmzA (Fig. 5, Fig. S1) shows that substantial changes take place in the bulge edge segment and the S1′-wall forming segments, effecting main-chain and side-chains of the corresponding residues. This is opening up the substrate-binding groove in order to provide space for a ligand. The side-chains of the conserved Phe80 and Phe82 residues move slightly while the side chain of Met78 adopts another conformation. In the S1′-wall forming segment the side chain of Phe136 performs a χ1 rotation of 115° and interacts with the malonate carboxylate group with its Cδ1 just 3.3 Å away from the malonate oxygen atom. The side chain of Asn138 follows this movement by occupying another rotamer while its main chain Cα moves by 2.1 Å. A similar effect was observed in the inhibitor-bound form of ADAM33, where the active site cleft widened through a 2 Å movement of the S1′-wall forming segment as a consequence of inhibitor binding [19]. Phenylalanine 136 is located in a position equivalent to the tyrosine residue found in other metzincin families that is considered to flip back and forth during catalysis and to be important for oxyanion stabilization and/or substrate release (also called the tyrosine switch) [15]. A phenylalanine side-chain does not provide the phenolic hydroxyl group, which is involved in the coordination of the tetrahedral addition-intermediate in the course of hydrolysis. Nevertheless, it could still play a role in substrate binding and/or the stabilization of the product amino group by forming cation-pi interactions as found in other metalloproteins [4], [26]. This phenylalanine residue is highly, although not strictly conserved within the archaemetzincin family [18].

#### S1′ subsite

Natural substrates of archaemetzincins are still unknown [12]. Examination of the nat-*Af*AmzA molecular surface reveals a small, closed S1′-specificity pocket, as found in many other metzincins, especially matrix metalloproteases MMP-1 and MMP-7 [27], [28]. Its depth is defined by Glu114 (on helix α2) and Lys146 (in specificity loop) that are located in positions equivalent to Leu218 in MMP-13 and Arg114 in MMP-1 [27], residues which define the size of their S1′-specificity pockets. The electrostatic surface potential of *Af*AmzA shows mainly acidic and hydrophobic residues in the substrate-binding groove (data not shown), hinting at a basic and hydrophobic substrate.

#### Cys_4_ zinc finger

The presence of the Cys_4_ zinc finger opens another – admittedly speculative – road to a possible regulation of archaemetzincins. The localization of these cysteines in the CTD (Fig. 2*C*) implies a possible regulatory mechanism for archaemetzincins, as the oxidation of the Cys_4_-Zn finger would lead to disulfide bond formation. The resulting conformational change could transform into the active site and influence the activity of archaemetzincins. Activation of proteins by oxidation of cysteines is a well-studied principle and was reported for e.g. Hsp33, where an oxidation of Cys_4_-Zn to disulfide bonds occurs [29]. On the other hand, the Cys_4_-Zn site could strictly serve the purpose of structural integrity.

In order to get some insight into the role of this structural element that is unique to archaemetzincins we prepared a mutant from the related *Methanopyrus kandleri* AmzA where Cys163 was replaced by alanine. This mutant protein expressed well but it could not be purified in a similar quality as the wild-type protein. Furthermore, the Cys163Ala variant did not show any proteolytic activity and precipitated within 12 hours after purification. Thus, we conclude that this is a structural metal site important for the stability of the folded state, similar to the disulfide bonds found at this position in other metzincins. Experiments on *M. xanthus* CarG variants support these findings, as proteins with single substitutions of all four cysteines in the cluster to serine failed to complement in ΔcarG mutant *M. xanthus* strains and preparation of an apo-CarG led to decreased stability and increased insolubility of the protein [15]. This led the authors to the conclusion that the conserved cysteines may play an important structural role as zinc ligands.

In summary, we report here the crystal structure of the archaemetzincin AmzA from *Archaeoglobus fulgidus* in different ligand-bound states at high resolution. Similar to a previous publication on a homolog from *Methanopyrus kandleri* [12], we observe a partially occluded active site cleft that may explain the proteolytic inactivity observed in our assays. On the other hand, we detect upon ligand binding opening of the active site at the S' sites. This allows the interpretation that archaemetzincins could be capable of catalyzing peptide bond hydrolysis under certain conditions. Aminopeptidase activity has been published for human AMZ1 and 2 [14], although this has to be confirmed.

## Materials and Methods

### Cloning, Expression and Purification

A synthetic DNA fragment coding for the full-length afamzA gene (UniProt ID O29917) and optimized for *E. coli* expression (MrGene) was cloned using the NdeI/XhoI restriction sites into the vector pET-28a (Novagen) for N-terminally 6xHis-tagged protein (NHis-AfAmzA) and pET-22b for untagged protein (nat-AfAmzA). Overexpression was carried out in E. coli BL21 (DE3) (Novagen) in LB medium for 4 h at 37°C. Nat-AfAmzA was purified by cation exchange chromatography and NHis-AfAmzA by immobilized metal affinity chromatography (IMAC, Ni-NTA). The 6xHis-tag was cleaved off by thrombin (1 U/mg protein, Sigma-Aldrich) and separated from the digested protein by IMAC, leaving a 3-residue overhang (GSH) at the N-terminus of NHis-AfAmzA. Both proteins were further purified by size exclusion chromatography (SEC) using a Superdex 75 16/60 column (GE Healthcare). Both AfAmzA constructs eluted from the SEC column in a monomeric state with a molecular weight of 18 kDa. Fractions containing AfAmzA were pooled and concentrated to 6 mg/ml.

The *Mk*AmzA Cys163Ala variant was prepared according to [30] and along with the wild-type protein purified as in [12].

### Crystallization and Structure Determination

Crystallization was carried out using the sitting-drop vapor diffusion method at 293 K in 25% PEG 3350, 0.2 M (NH_4_)_2_SO_4_, 0.1 M HEPES pH 7.5 (NHis-*Af*AmzA), 12% PEG 3350, 0.1 M sodium malonate pH 6.0 (nat-*Af*AmzA::malonate) and 0.9 M ammonium sulfate, 0.1 M citric acid pH 4.0 (nat-*Af*AmzA::citrate) at a protein concentration of 6 mg/ml. For data collection crystals were cryo-protected with 37.5% PEG 3350 (or 20% glycerol for nat-*Af*AmzA::citrate) and flash-cooled in liquid nitrogen. Diffraction data were collected under cryogenic conditions on beamline X06DA at the Swiss Light Source (PSI, Villigen, Switzerland) at a wavelength of 1.00 Å using a MAR225 detector. Data were indexed, processed, and scaled using the XDS software package (Kabsch, 2010). The structure of NHis-AfAmzA was solved by molecular replacement using the structure of Methanopyrus kandleri AmzA (PDB ID 2X7M, 49% sequence identity) [12] and of nat-AfAmzA using the NHis-AfAmzA structure (PDB ID 4AXQ) with the program Phaser [31], [32]. Structure refinement and model building was performed using iterative cycles of phenix.refine [33] and Coot [34]. The TLS Motion Determination (TLSMD) server [35] and phenix.refine were used in order to determine the optimal number of TLS groups. We further attempted full anisotropic a.d.p. refinement for the tetragonal and triclinic crystal form. In the tetragonal crystal form this lowered Rwork and Rfree by 1.3% and 0.3%, respectively. Similar results were obtained in the triclinic crystal form. Therefore, individual anisotropic a.d.p. refinement appears as not justified. Data collection and refinement statistics are summarized in Table 1. Figures were prepared using PyMOL (http://www.pymol.org).

#### Accession codes

The atomic coordinates and structure factors have been deposited in the Protein Data Bank [36] with accession codes 4AXQ (NHis-*Af*AmzA), 3ZVS (nat-*Af*AmzA::malonate) and 4A3W (nat-*Af*AmzA::citrate).

## Supporting Information

Figure S1 **Main chain RMS deviation of the three** ***Af*** **AmzA structures.** RMS deviation plots for the main chain atoms of the apo and ligand bound structures. The black line highlights the mean RMSD of 0.4 Å. Compared to NHis-*Af*AmzA residue Asn138 located in the specificity loop shows a RMSD of 1.6 Å (nat-*Af*AmzA::malonate, dotted red line) and 1.8 Å (nat-*Af*AmzA::citrate, solid blue line), respectively.
(TIF)

Figure S2 **Overlay of** ***M. kandleri*** **AmzA in non-liganded and citrate-bound form.** The non-liganded form is depicted in cyan, the citrate-bound form as red ribbon. Citrate is shown as sticks with orange carbon atoms and red oxygens.
(TIF)
