# Supplementary figures and images for: Crystal Structures of Archaemetzincin Reveal a Moldable Substrate-Binding Site
Source: PLoS One. 2012 Aug 24;7(8):e43863. doi: 10.1371/journal.pone.0043863 (PMC3427221; doi:10.1371/journal.pone.0043863)

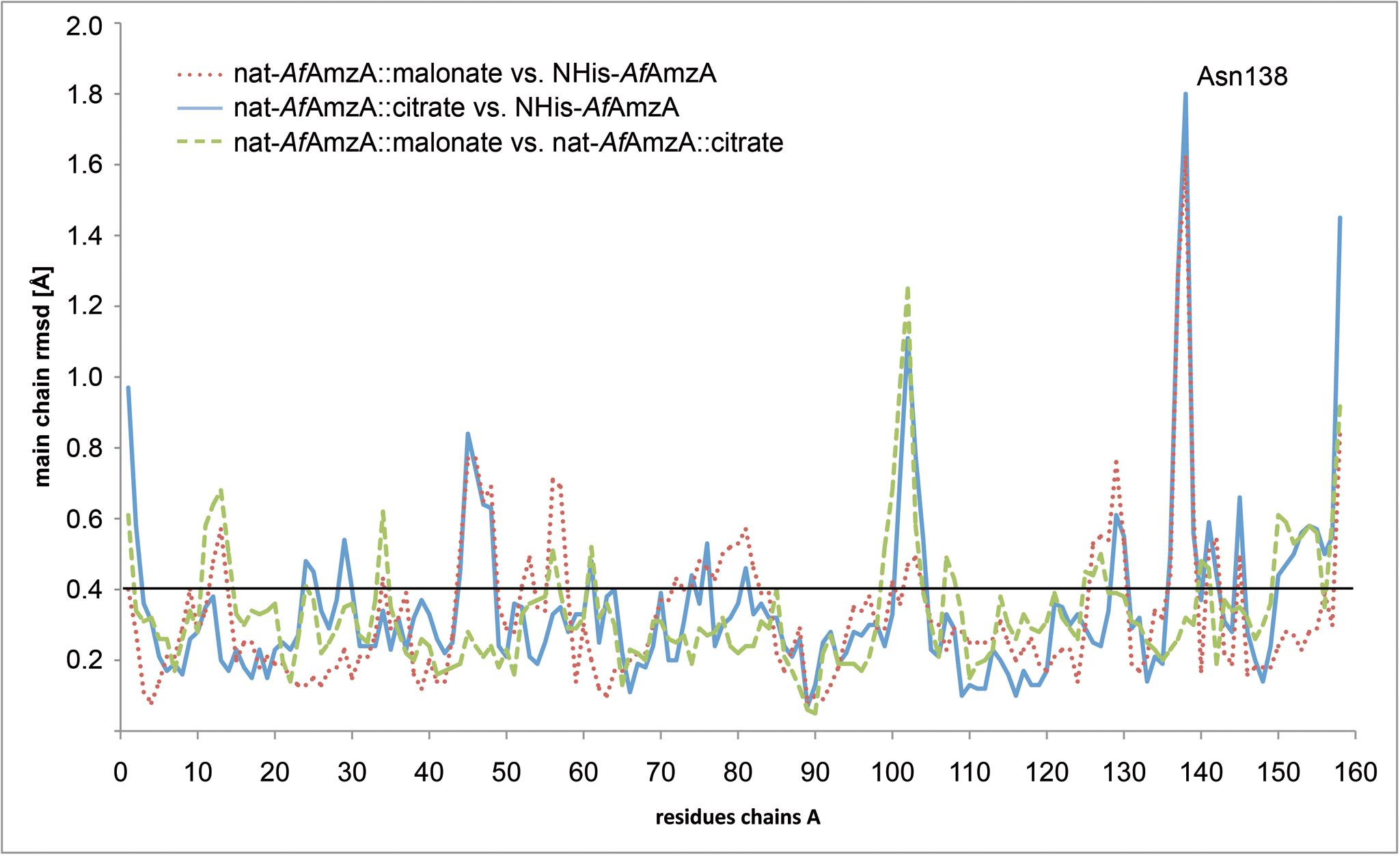

Supplement: Figure S1 — Main chain RMS deviation of the three Af AmzA structures. RMS deviation plots for the main chain atoms of the apo and ligand bound structures. The black line highlights the mean RMSD of 0.4 Å. Compared to NHis-AfAmzA residue Asn138 located in the specificity loop shows a RMSD of 1.6 Å (nat-AfAmzA::malonate, dotted red line) and 1.8 Å (nat-AfAmzA::citrate, solid blue line), respectively. (TIF) [file pone.0043863.s001.tif]

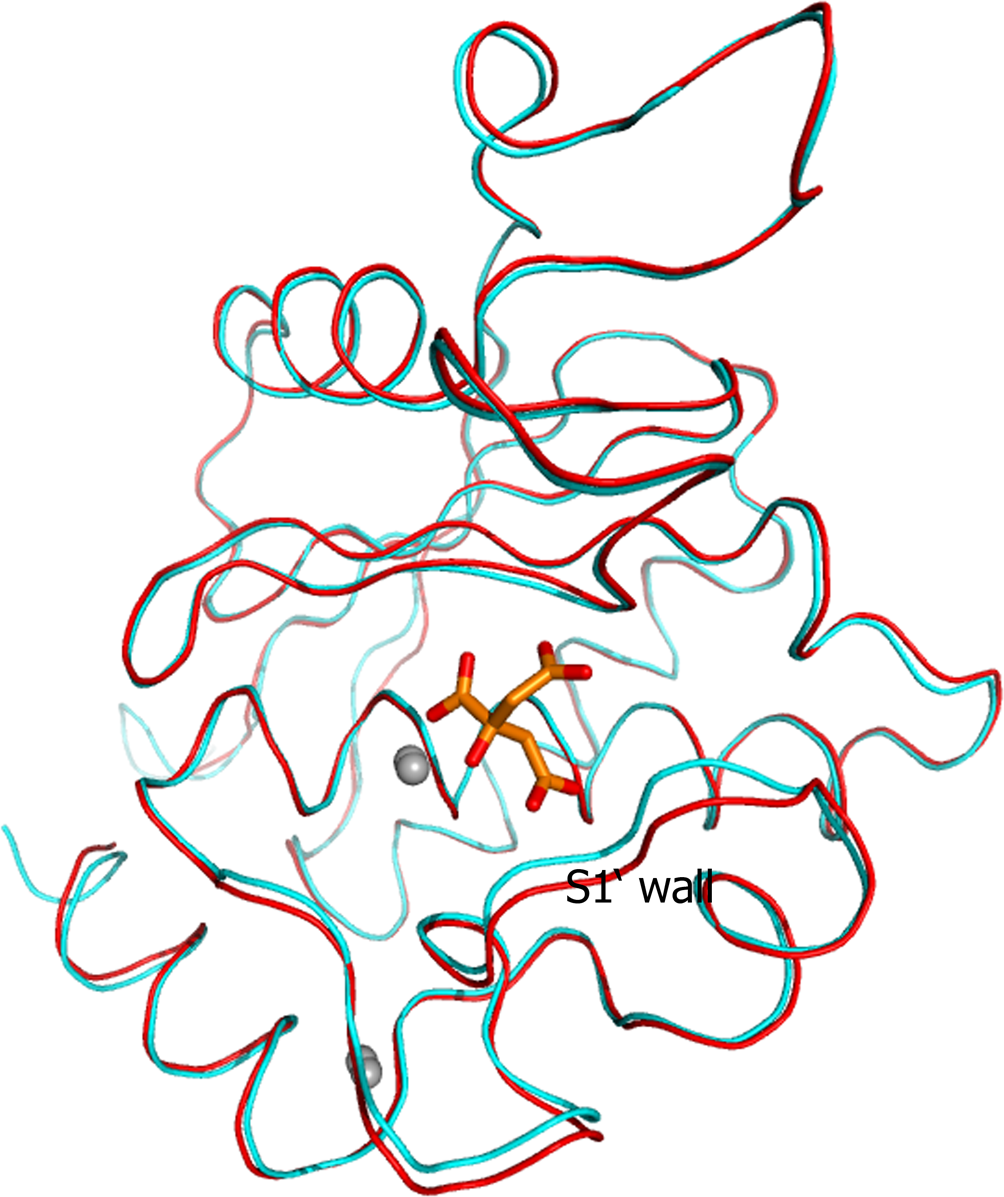

Supplement: Figure S2 — Overlay of M. kandleri AmzA in non-liganded and citrate-bound form. The non-liganded form is depicted in cyan, the citrate-bound form as red ribbon. Citrate is shown as sticks with orange carbon atoms and red oxygens. (TIF) [file pone.0043863.s002.tif]
